# Dr. James J. Bowen

**Published:** 1916-03

**Authors:** 


**Dr. James J. Bowen,** N. Y .Univ. 1895, died at Saranac Lake, Jan. 12, aged 40.

